# A Surgical Case of an Abdominal Aortic Aneurysm With a Meandering Inferior Mesenteric Artery due to Superior Mesenteric Artery Occlusion

**DOI:** 10.1155/crvm/7322019

**Published:** 2025-05-09

**Authors:** Shun Hiraga, Takehisa Abe, Ryohei Fukuba, Junichi Takemura, Rei Tonomura, Sayaka Tamada, Kazuhiro Mitani, Mitsuharu Hosono

**Affiliations:** Department of Thoracic and Cardiovascular Surgery, Nara Medical University, Kashihara, Nara, Japan

## Abstract

A 78-year-old man was diagnosed with an abdominal aortic aneurysm with a meandering mesenteric artery. We performed abdominal aortic replacement and inferior mesenteric artery reconstruction using intraoperative inferior mesenteric artery perfusion during surgery. A 4-Fr arterial sheath was inserted into the left brachial artery, and a 10-Fr balloon catheter was inserted into the inferior mesenteric artery for perfusion. The intraoperative intestinal blood flow was satisfactory, and the patient's postoperative course was favorable. This method was an easy and effective option for abdominal aortic surgery in patients with a meandering mesenteric artery.

## 1. Introduction

There have been case reports of abdominal aortic aneurysms with abdominal aortic branch occlusions, such as the celiac and superior mesenteric arteries, in which the inferior mesenteric artery was an important collateral channel for intestinal blood flow. Occlusion of the superior mesenteric artery may cause enlargement of the Riolan arch, which is called the meandering mesenteric artery [1]. In a previous report of a case of abdominal aortic surgery with a meandering mesenteric artery [2], abdominal aortic occlusion under the renal artery caused severe intestinal ischemia resulting in a fatal outcome due to interruption of intestinal blood flow. Thus, the maintenance of meandering mesenteric arterial flow is important in abdominal aortic surgery in such patients. Herein, we report a successful surgical case of an abdominal aortic surgery using an external shunt to perfuse the meandering mesenteric artery in a patient with an occlusion of the superior mesenteric artery.

## 2. Case Presentation

**Figure 1:**
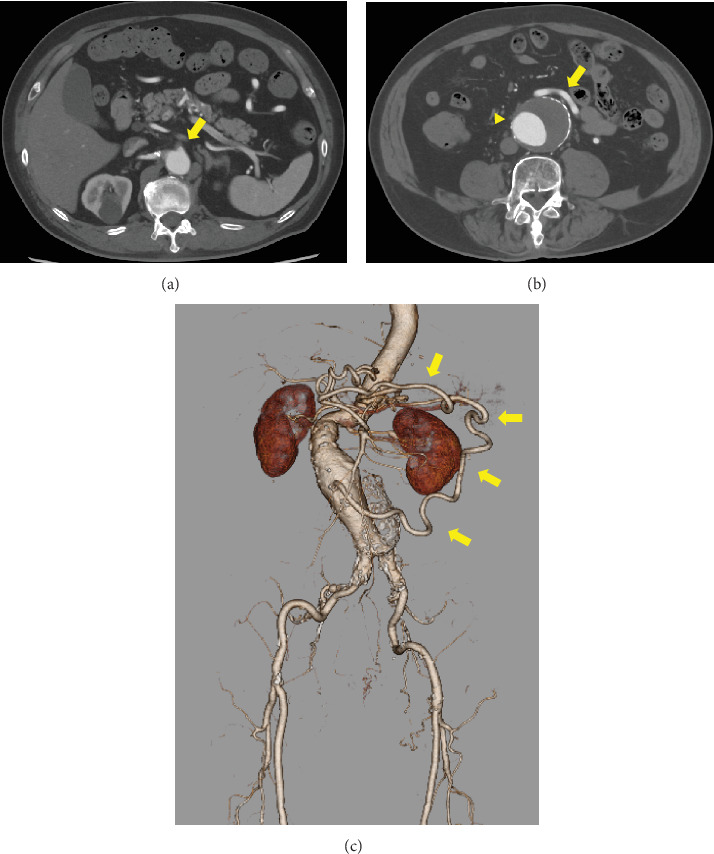
(a) A preoperative contrast-enhanced computed tomography (CT) revealing occlusion of the superior mesenteric artery (arrow). (b) The diameter of the abdominal aortic aneurysm and the inferior mesenteric artery is 53 mm (arrowhead) and 8 mm (arrow), respectively. (c) A multiplanner reconstruction of CT revealing the dilated arch of Riolan (arrows).

A 78-year-old man was referred to our hospital for further evaluation of a 1-cm nodule in the right lung, observed on computed tomography (CT). The patient had a history of hypertension, hyperuricemia, ischemic heart disease, pulmonary fibrosis complicated by emphysema, and esophageal hernia. The pulmonary nodule was diagnosed as an inflammatory nodule after close examination; however, CT revealed an abdominal aortic aneurysm measuring 54 mm in diameter. The patient was then referred to our department. His physical examination was normal except for a pulsatile mass on the lower abdomen. There was no recent weight loss, abdominal pain, bleeding, or other symptoms suggestive of intestinal ischemia. A blood test revealed a mildly elevated white blood cell count (9200/*μ*L) and C-reactive protein levels (0.12 mg/dL). Mildly prolonged activated partial thromboplastin time (27.9 s) and a mildly elevated D-dimer level (2.7 mg/dL) were also observed. Contrast-enhanced CT revealed an infrarenal abdominal aortic aneurysm with a maximum diameter of 54 mm. The superior mesenteric artery was occluded at its origin, the inferior mesenteric artery was enlarged to 8 mm in diameter, and the Riolan arch was enlarged, leading to the diagnosis of a meandering mesenteric artery (Figure 1). Thus, the inferior mesenteric artery was enlarged as a collateral blood source for intestinal blood flow. From the results of these findings, we planned the reconstruction of the inferior mesenteric artery to avoid intestinal ischemia and abdominal aortic replacement.

**Figure 2:**
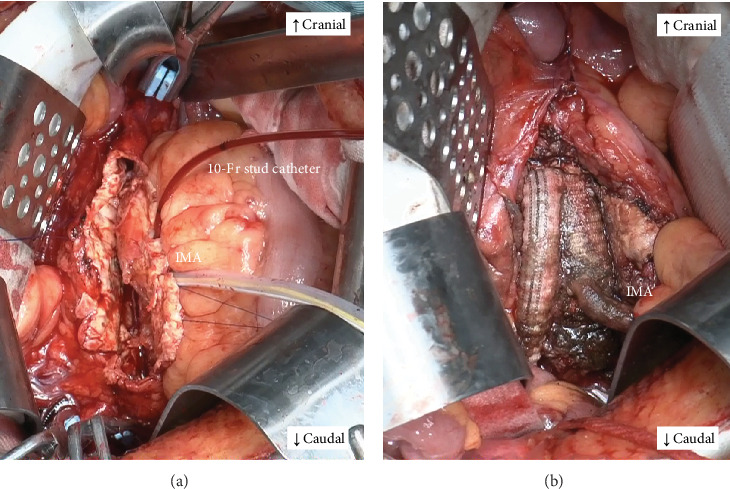
(a) Intraoperative view of the case. A 10-Fr cannula is inserted into the inferior mesenteric artery (IMA). (b) After the reconstruction of the IMA.

**Figure 3:**
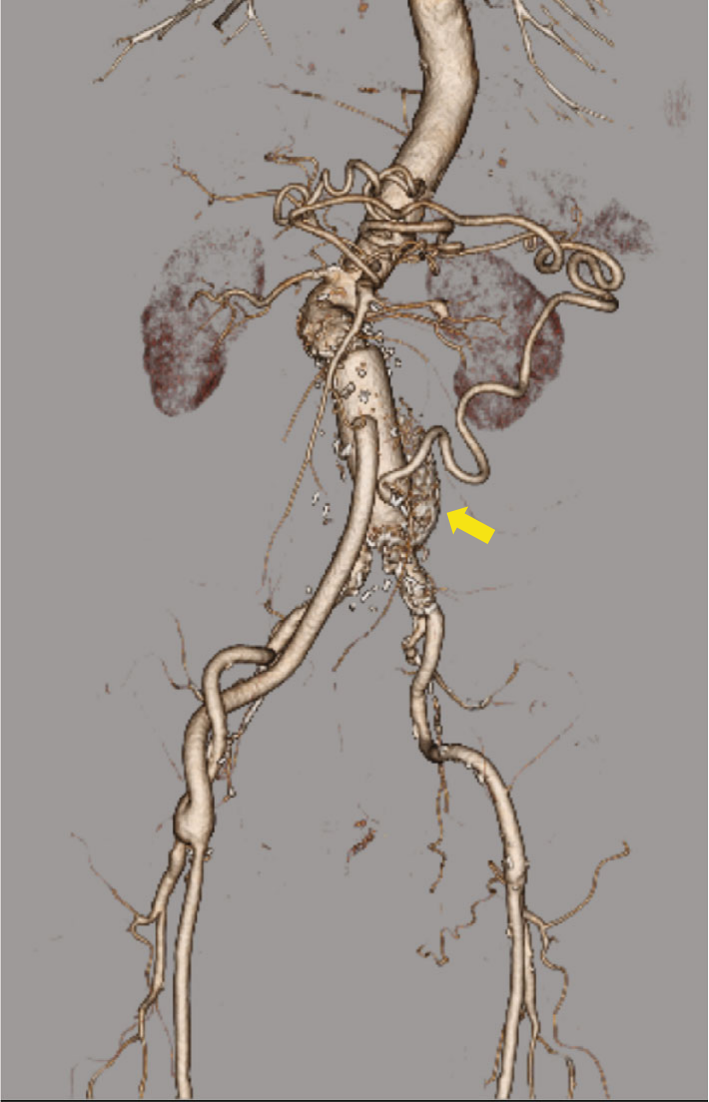
Postoperative contrast-enhanced computed tomography reveals the patent inferior mesenteric artery (arrow).

Under general anesthesia, a 4-Fr short arterial sheath was placed into the left brachial artery in advance. Due to the maintenance of the intraoperative intestinal blood flow through the inferior mesenteric artery during abdominal aortic clamping, we planned to perfuse the inferior mesenteric artery using an external shunt. A median abdominal incision was made, and dissection with mild adhesions was performed to reach the intraperitoneal cavity. After intravenous administration of 5000 units of heparin, the abdominal aorta was clamped under the bilateral renal and common iliac arteries. The inferior mesenteric artery was clamped using a tourniquet. The aneurysm was incised, and some lumbar arteries were closed with 3–0 polypropylene from inside the aorta. A 10-Fr balloon catheter (Sumitomo Bakelite, Tokyo, Japan) was inserted into the inferior mesenteric artery and connected to a sheath placed in the left brachial artery to perfuse the inferior mesenteric artery using an external shunt (Figure 2a), and proximal anastomosis of aortic reconstruction was performed. The color tone of the intestine was adequate after the proximal anastomosis, and there was no problem palpating the beating of the mesenteric vessels. Therefore, we decided to perform a distal anastomosis prior to the reconstruction of the inferior mesenteric artery, considering the advantage of easy positioning of the reconstruction site. After completion of Y-grafting, the balloon catheter in the inferior mesenteric artery was removed, and the external shunt ceased. The perfusion time was 80 min. The inferior mesenteric artery was reconstructed by anastomosing it onto the left leg of the graft using continuous 6–0 polypropylene sutures (Figure 2b). Because the right femoral artery was not palpable after releasing the clamp, we performed a bypass to the right common femoral artery using an 8-mm vascular graft. Right femoral artery blood flow was palpable after the bypass. Intraoperative intestinal coloration was adequate throughout the surgery, and vascular pulsation of the mesentery was well palpable. The operation time was 6 h and 34 min, with a blood loss of 645 mL. The patient resumed eating on the second postoperative day. Postoperatively, there were no abdominal symptoms suggestive of intestinal ischemia. A contrast-enhanced CT scan performed on the 14th postoperative day showed good blood flow in the inferior mesenteric artery and no blood flow disturbance in the gastrointestinal tract (Figure 3). The patient was discharged ambulatory on the 17th postoperative day. Currently, 6 months after surgery, the patient is under outpatient observation, with no particular problems. Consent was obtained from the patient to publish this case report.

## 3. Discussion

There have been reports of abdominal aortic aneurysms complicated by the occlusion of branch arteries such as the celiac, superior mesenteric, and inferior mesenteric arteries. In a report examining 713 cases of abdominal aortography, stenosis was found in 12.5% of celiac artery branches and 3.4% of superior mesenteric artery branches [3]. In another report of aortography in a group of 205 patients with aortic aneurysms or leg occlusive arterial disease, stenotic lesions were found in the celiac artery in 25% of patients, the superior mesenteric artery in 6%, and in both the celiac and superior mesenteric arteries in 3.4% of patients [4]. When there is severe stenosis or occlusion of the visceral arteries (celiac, superior mesenteric, and inferior mesenteric arteries) and abdominal aorta, the anastomotic artery between the three visceral arteries abnormally dilates to form collateral blood vessels. Moskowitz et al. named the abnormally dilated, meandering central anastomotic artery of the colon as the meandering mesenteric artery [1]. In our case, a meandering mesenteric artery due to superior mesenteric artery occlusion was observed on the preoperative CT. In cases of meandering mesenteric arteries, the pancreaticoduodenal, Bühler, and colonic marginal (Drummond's artery) arteries and the Riolan's arch can be identified as collateral blood vessels. In our case, the Riolan's arch was enlarged.

In patients with abdominal aortic aneurysms with a meandering mesenteric artery, which develops as an important collateral blood source, intestinal ischemic complications must be considered during aortic surgery. Intestinal ischemia is one of the crucial complications of abdominal aortic surgery, and it often leads to fatal outcomes. In a review of more than 6300 cases in 20 reports of visceral infarction following aortic surgery, colonic ischemia was found in 1.5% of all patients after abdominal aortic reconstructive surgery and small bowel ischemia in 0.15%, with mortality rates of 40% and 90%, respectively [5]. In order to avoid colon ischemia, the inferior mesenteric artery should be reconstructed in abdominal aortic surgery for patients with visceral artery occlusion. Rogers et al. also showed improvement in prognosis after aortic surgery by prophylactic mesenteric revascularization in a report on the treatment of patients with mesenteric blood flow disorders [2]. As another possible alternative, endovascular treatment can be used for mesenteric revascularization. Sharafuddin et al. [6] reported that two patients with postprandial abdominal pain who underwent stenting of the celiac artery or superior mesenteric artery before abdominal aortic surgery to develop collateral vessels had adequate results. However, in a comparative study between endovascular treatment and laparotomy in patients with chronic intestinal ischemia, the symptom recurrence rate was significantly higher in the endovascular treatment group [7]. Furthermore, in a report of patients with occlusion of the celiac artery, superior mesenteric artery, and inferior mesenteric artery origin who underwent endovascular repair for abdominal aortic aneurysm, median laparotomy was performed for mesenteric artery bypass [8]. For these reasons, we adopted open surgery for abdominal aortic aneurysm in this case.

In any case, in the presence of occlusion of the celiac and superior mesenteric arteries, the inferior mesenteric artery is likely to develop as an important collateral blood source; it is crucial to maintain intestinal blood flow and reconstruct the inferior mesenteric artery. In a previously reported case of abdominal aortic aneurysm surgery complicated by superior mesenteric artery occlusion, a 12-Fr catheter was connected after the anastomosis of an 8-mm expanded polytetrafluoroethylene prosthetic graft to the right subclavian artery, and the inferior mesenteric artery was perfused with an external shunt [9]. However, this method was expensive since a prosthetic graft was used, and an additional wound was necessary for the subclavian artery. In another case report, an 8-Fr sheath was inserted into the abdominal aortic neck on the proximal side to provide an external shunt to the inferior mesenteric artery [10]. However, in this method, the abdominal aortic neck was crowded at the proximal clamping site. In our present case, an external shunt was established between a 4-Fr short sheath in the left brachial artery and a 10-Fr balloon catheter in the inferior mesenteric artery to perfuse the inferior mesenteric artery with an external shunt. This method was simple and easy to perform. A bypass to the superior mesenteric artery is a possible approach, but it is important to ensure superior mesenteric artery blood flow during the abdominal aortic clamp. For this purpose, the bypass inflow should be farther from the proximal abdominal clamp. It is relatively difficult to further expose the central aorta through a median abdominal incision approach. We considered this method, which can be performed in the same field of view, to be easy and safe.

In conclusion, although further follow-up is necessary to evaluate the symptoms and presence of arterial enlargement, the method used in our case was simple and easy to perform. This method can be an effective option for abdominal aortic aneurysm surgery complicated by a meandering mesenteric artery.

## Data Availability

Data sharing is not applicable to this article as no new data were created or analyzed in this study.
